# Identification of cuproptosis-related genes in septic shock based on bioinformatic analysis

**DOI:** 10.1371/journal.pone.0315219

**Published:** 2024-12-09

**Authors:** Jintong Zhao, Meng Zhang, Ying Wang, Feifei He, Qiang Zhang

**Affiliations:** 1 Department of Critical Medicine, Zibo Central Hospital, Zibo, China; 2 Department of Critical Medicine, Qingdao Central Hospital, Qingdao, China; 3 Department of Nosocomial Infection, Qingdao Cancer Hospital, Qingdao, China; 4 Department of Critical Medicine, Qingdao Hiser Hospital, Affiliated Hospital of Qingdao University (Qingdao Traditional Chinese Medicine Hospital), Qingdao, China; Children’s National Hospital, George Washington University, UNITED STATES OF AMERICA

## Abstract

**Background:**

Septic shock is a life-threatening condition characterized by a failure of organ systems and a high mortality rate. Cuproptosis is a new form of cell death that is triggered by copper overload. However, the relationship between cuproptosis-related genes and septic shock remains unclear.

**Methods:**

The GSE26440 dataset from the GEO database was used to screen differentially expressed genes (DEGs) between control and septic shock samples. Additionally, hub genes related to the progression of septic shock and cuproptosis were screened by Venn analysis. RT-qPCR was utilized to validate the expression of hub genes in peripheral blood lymphocytes from septic shock patients and healthy controls. Next, functional analysis and immune cells infiltration were performed.

**Results:**

SLC31A1 and MTF1 levels were obviously elevated and LIAS and LIPT1 levels were downregulated in septic shock samples, compared to normal controls. The diagnostic values of the four genes were confirmed with receiver operating characteristic (ROC) curves. Additionally, SLC31A1 and MTF1 showed a positive correlation with natural killer cells and LIAS and LIPT1 exhibited a positive correlation with CD8+ T cells. Furthermore, compared to low-level groups, MAPK signaling was activated in the high-SLC31A1 level group, VEGF signaling was activated in the high-MTF1 level group and lipoic acid metabolism was activated in high-LIAS and high-LIPT1 level groups.

**Conclusion:**

This study demonstrates that SLC31A1, MTF1, LIAS, and LIPT1 are dysregulated in septic shock samples, and these genes exhibit potential diagnostic efficacy in septic shock, suggesting that these genes may be potential biomarkers for the diagnosis of septic shock.

## 1. Introduction

Sepsis is a serious organ dysfunction syndrome caused by an imbalance in the body’s response to infection [1]. It is a main cause of morbidity and mortality among hospitalized patients in the world [2]. Septic shock represents the most severe complication of sepsis [3] and accounts for a major cause of death in intensive care unit (ICU). [4]. Septic shock is characterized by fluid-refractory hypotension (mean blood pressure <60 mmHg) and persistent hyperlactatemia (blood lactate concentrations > 4 mmol/L) [5,6]. Thus, modulation of the host response (e.g. vasopression, hydrocortisone and blood purification), haemodynamic stabilization (intravenous fluids) and infection control (e.g. antibiotics) are three main branches of septic shock treatment [7]. Despite these interventions, research indicates that the in-hospital mortality rate for patients with septic shock exceeds 30% [8]. Evidence suggests that blood cultures and PCR assays for detecting the causative microorganisms, such as bacteria or viruses, are critical for the early diagnosis of infectious diseases [9,10]. Early diagnosis facilitates timely treatment and improves patient outcomes in septic shock [11]. Therefore, there is an urgent need for the development of new biomarkers for the early diagnosis of septic shock.

Copper is an essential trace element in the body, which plays a crucial role in maintaining essential cellular functions under physiological concentrations [12]. However, excess copper can cause cellular toxicity, ultimately leading to cell death [13,14]. Moreover, excess copper can trigger inflammation and oxidative stress, contributing to tissue damage [15]. Evidence have shown that the dysregulation of copper homeostasis is associated with multiple diseases including neurological disorders [16], cardiovascular diseases [17], cancers [18] and sepsis [19]. Zhang et al. found that the concentration of copper in the whole blood of sepsis patients was higher than that in healthy controls [20]. Xu et al. reported that compared to sepsis patients in the survival group, whole blood copper levels were notably elevated in the death group of sepsis patients [19], suggesting that high copper levels are associated with the poor prognosis in sepsis patients. These data showed that the copper homeostasis was dysregulated in sepsis.

Cuproptosis is a novel form of cell death that is triggered by copper overload [21]. It has been shown that cuproptosis plays a significant role in the pathogenesis of human diseases, such as neurological disorders [22], cardiovascular diseases [23], rheumatoid arthritis [24], cancers [25]. However, the involvement of cuproptosis in septic shock remains largely unstudied. Therefore, we aimed to identify cuproptosis-related genes that may be involved in septic shock based on the data in GEO databases. Our results showed that cuproptosis-related genes (e.g. SLC31A1, LIAS, MTF1, LIPT1) were obviously dysregulated in septic shock. These genes may predict the risk of septic shock and may serve as potential biomarkers for the prevention and treatment of septic shock in the future.

## 2. Materials and methods

### 2.1 Data collection

The datasets GSE26440 (including 98 septic shock samples and 32 normal controls), GSE33118 (including 20 septic shock samples and 42 normal controls), GSE236713 (including 200 pulmonary sepsis samples and 30 normal controls) and GSE57065 were downloaded from the Gene Expression Omnibus (GEO, http://www.ncbi.nlm.nih.gov/geo/). Meanwhile, mRNA, miRNA and lncRNA in the GSE57065 dataset were identified based on the annotation information obtained from the NCBI database (GCF_000001405.38_GRCh38.p12_genomic). 19 cuproptosis-related genes were listed in S1 Table [26].

### 2.2 Differential expressed analysis

The “limma” R package (version 3.52.4) was applied to identify differentially expressed genes (DEGs) between septic shock samples and normal controls with the criteria of |log2FC| > 2 and p.adjust < 0.05 [27].

### 2.3 Drug prediction

CMap database (https://clue.io) allows users to explore the networks of drugs, small molecule compounds, genes, and disease states on the L1000 analysis platform, which is based on data from 164 drugs/small molecule compounds and cell expression profiles processed using overexpression or gene knockout tools. Based on the median expression levels of each gene [SLC31A1 (copper transporter 1), LIAS (lipoic acid synthase), MTF1 (metal-responsive transcription factor 1) or LIPT1 (lipoyltransferase 1)], samples was grouped into high-level and low-level groups respectively. DEGs were screened between two groups. Then, drug prediction was conducted using top 50 upregulated and top 50 downregulated genes.

### 2.4 Functional analyses

Gene Ontology [GO, including biological process (BP), molecular function (MF) and cellular component (CC)] and Kyoto Encyclopedia of Genes and Genome (KEGG) enrichment analyses were conducted using the “clusterProfiler” R package (version 4.7.1.2) with the criteria of p < 0.05 [28].

Gene set variation analysis (GSVA) was performed using the “c2.cp.kegg.v2023.1.Hs.symbols” and “c5.go.v2023.1.Hs.symbols” datasets, which were downloaded from the Molecular Signature Database (https://www.gsea-msigdb.org/gsea/msigdb). The significant pathways were identified based on the criteria of |logFC| < 0.5 and p.adjust < 0.05.

Based on the median expression levels of each gene (SLC31A1, LIAS, MTF1 or LIPT1), samples was grouped into high-level group and low-level group respectively. DEGs were screened between two groups. Gene Set Enrichment Analysis (GSEA) was performed on these DEGs with the criteria of p < 0.05.

### 2.5 Immune cell infiltration estimation with CIBERSORT

The relative levels of 22 immune cell types were evaluated using CIBERSORT software [29]. Based on the gene expression matrix, CIBERSORT software can analyze the composition of immune-infiltrating cells using the deconvolution algorithm. The sum of the proportions of all estimated immune cell types in each sample is equal to 1.

### 2.6 Construction of gene-miRNAs-lncRNAs network and gene-transcription factors (TFs) network

The correlation coefficients between hub genes and miRNAs, lncRNAs or TFs was calculated respectively. The p < 0.05 was set as the threshold to screen miRNAs and lncRNAs that were significantly correlated to hub genes. The p < 0.05 and |rho| > 0.4 were set as the thresholds to screen TFs that were significantly correlated to hub genes. Next, the hub gene-miRNAs-lncRNAs network and gene-TFs network were constructed.

### 2.7 Clinical samples

In total, 12 patients with septic shock and 12 healthy individuals were included in this study. Septic shock was diagnosed according to the Third International Consensus Definitions for Sepsis and Septic Shock (Sepsis 3.0) criteria [30]. The clinical information of all participants is presented in S2 Table. Blood samples were collected from each participant. Informed consent form was signed by all participants included in the study. The study received approval from the Ethics Committee of the ZIBO Central Hospital (No. IEC-form-030-2.0).

### 2.8 Real-time reverse transcriptase-polymerase chain reaction (RT-qPCR)

The peripheral blood lymphocytes were isolated from the blood samples using the human peripheral blood lymphocyte isolation fluid (LTS1077, TBD Science), and then total RNA was isolated from lymphocytes using the Blood Total RNA (miRNA) Extraction Kit (No. TSR4201-50, Beijing Tsingke Biotech Co., Ltd.). A SynScript ^TM^III cDNA Synthesis Mix kit (No. TSK322S, Beijing Tsingke Biotech Co., Ltd.) was used for reverse transcription. Real time PCR was conducted with the ArtiCanATM SYBR qPCR Mix kit (No. TSE501, Beijing Tsingke Biotech Co., Ltd.) on a fluorescence quantitative PCR instrument (QuantStudio 5, Life Technologies). The housekeeping gene β-actin was used as the reference gene for quantification. The 2^-ΔCt^ method was applied to calculate the relative expression of each gene [31]. β-actin: forward, 5’-CTCCATCCTGGCCTCGCTGT-3’ and reverse, 5’-GCTGTCACCTTCACCGTTCC-3’; MTF1: forward, 5’-CACAGTCCAGACAACAACATCA-3’ and reverse, 5’-GCACCAGTCCGTTTTTATCCAC-3’; LIAS: forward, 5’-CAGCCCAGTCAGACCGTTAAG-3’ and reverse, 5’-TTTCTGGCGTTTTAGGTTTCCT-3’; LIPT1: forward, 5’-TTGCTAAAGAGCCCTTACCAAG-3’ and reverse, 5’-TCATCCGTTGGGTTTATTAGGTG-3’; SLC31A1: forward, 5’-GGGGATGAGCTATATGGACTCC-3’ and reverse, 5’-TCACCAAACCGGAAAACAGTAG-3’;

### 2.9 Statistical analysis

Wilcoxon rank sum test was performed to compare the difference in gene expression and immune cell infiltration between two groups. Pearson correlation analysis was used to calculate the correlation coefficients using the “cor” R package. The receiver operating characteristic (ROC) curve was created using the “pROC” R package to verify the diagnostic value of each hub gene. For RT-qPCR analysis, differences between groups were analyzed by unpaired Student’s t-test. The statistical significance was set at p< 0.05.

## 3. Results

### 3.1 Screening of cuproptosis-related genes related to septic shock progression

**Fig 1:**
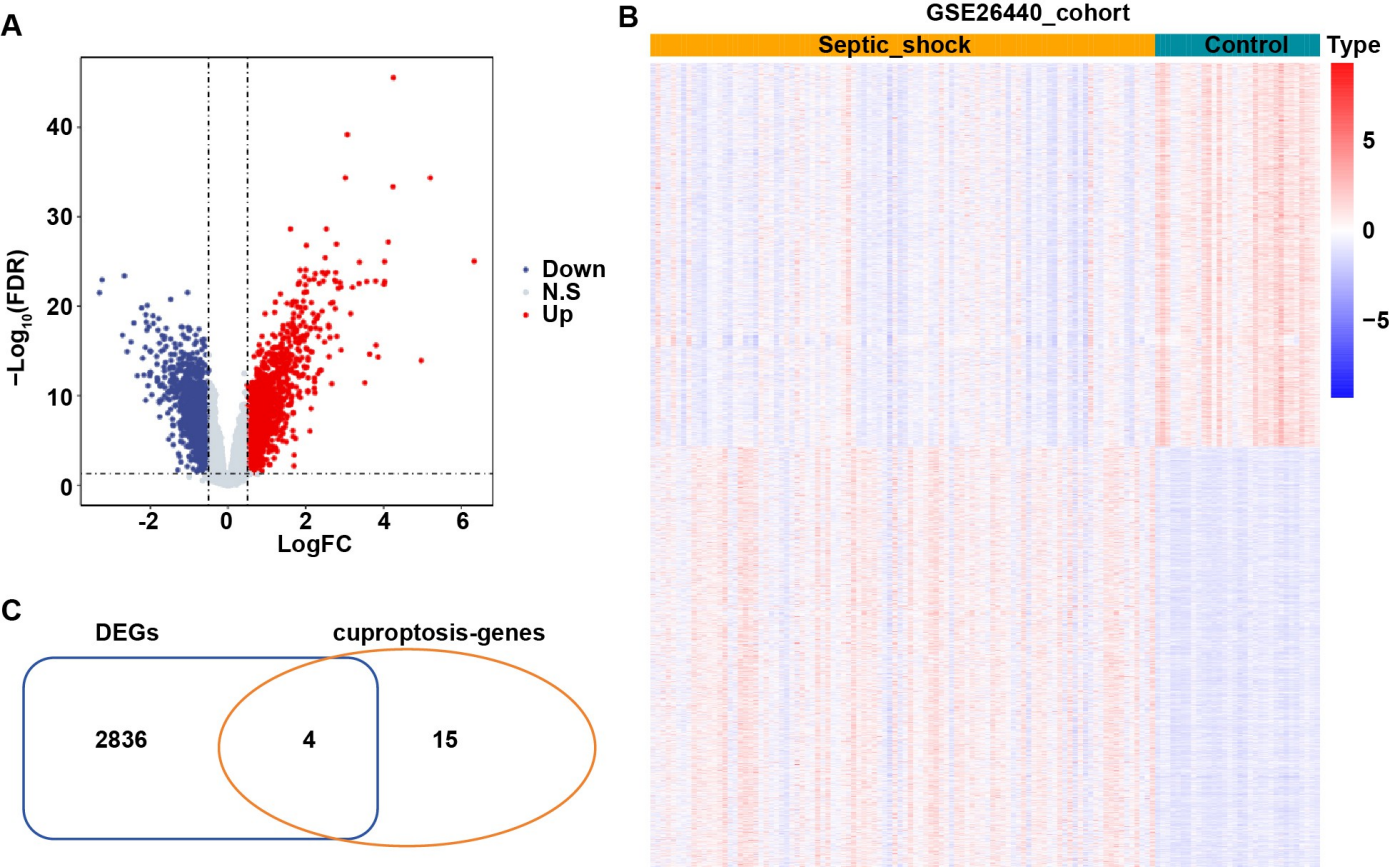
Screening of cuproptosis-related genes related to septic shock progression. **(A)** The volcano plot and **(B)** heat map displayed the DEGs between control and septic shock groups. Blue, downregulated genes; Red, upregulated genes. **(C)** The overlapping genes between 19 cuproptosis-related genes and DEGs were screened using the Venn map.

The “limma” R package was used to identify DEGs between control and septic shock groups based on data from the GSE26440 cohort. As shown in Fig 1A and 1B, compared to control group, a total of 2840 DEGs, including 1485 upregulated and 1355 downregulated genes, were identified in the septic shock group. Furthermore, 4 overlapping genes (SLC31A1, MTF1, LIAS and LIPT1) were identified between 19 cuproptosis-related genes and 2840 DEGs (Fig 1C), which were used as hub genes related to cuproptosis and septic shock progression. The expression distribution of these four genes in human tissues was obtained from The Human Protein Atlas (HPA) database and presented in S1A–S1D Fig.

### 3.2 The diagnostic value of hub genes in predicting septic shock

**Fig 2:**
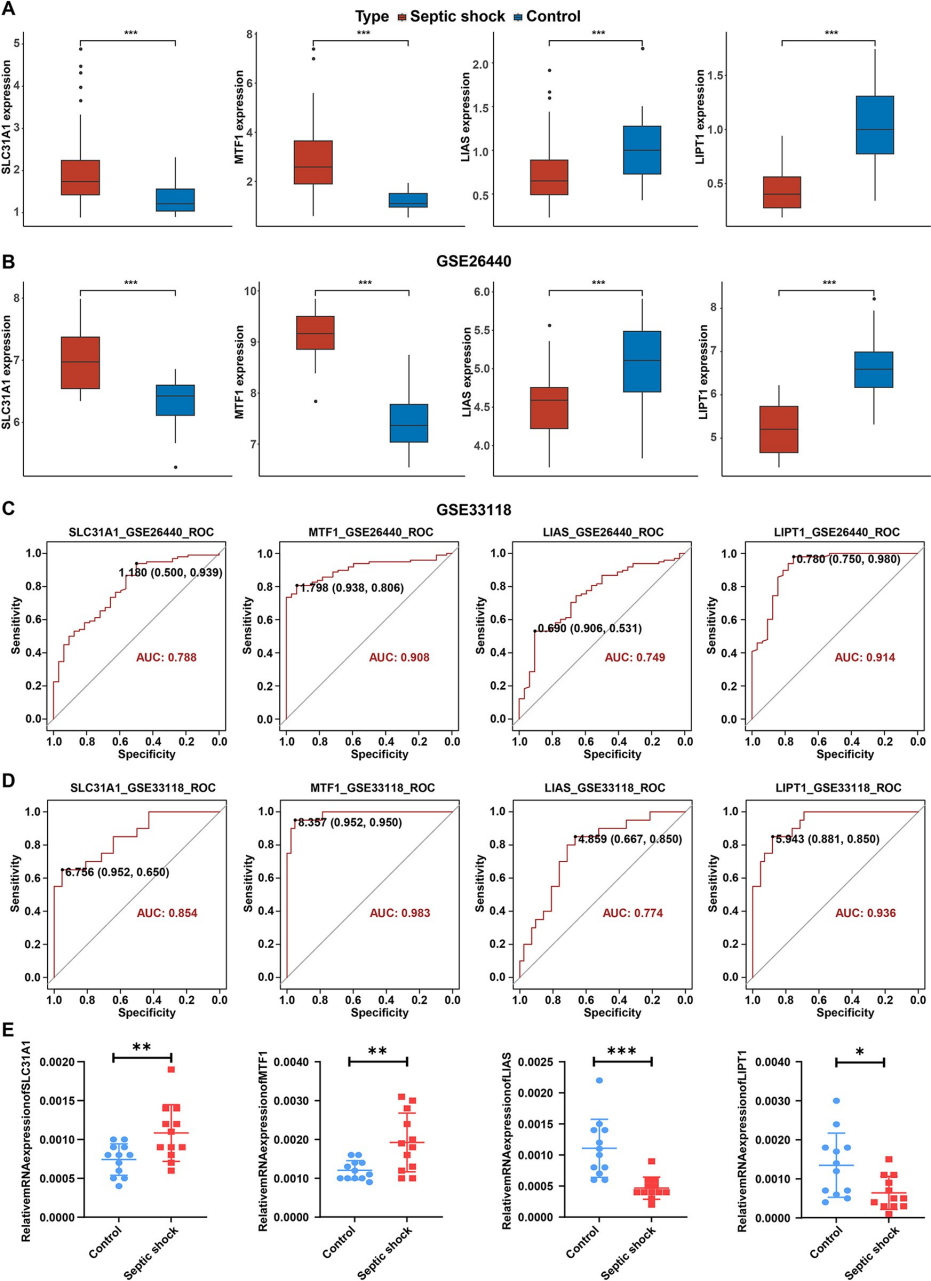
The diagnostic value of hub genes in predicting septic shock. **(A)** The Box plots displayed SLC31A1, MTF1, LIAS and LIPT1 levels between control and septic shock groups in the GSE26640 cohort. **(B)** The Box plots displayed SLC31A1, MTF1, LIAS and LIPT1 levels between control and septic shock groups in the GSE33118 cohort. **(C)** ROC curves verified the diagnostic value of SLC31A1, MTF1, LIAS and LIPT1 genes in the GSE26640 cohort. **(D)** ROC curves verified the diagnostic value of SLC31A1, MTF1, LIAS and LIPT1 genes in the GSE33118 cohort. **(E)** RT-qPCR analysis of SLC31A1, MTF1, LIAS and LIPT1 levels in peripheral blood lymphocytes from healthy controls and patients with septic shock. *P<0.05, **P<0.01, ***P<0.001.

In the GSE26440 cohort, SLC31A1 and MTF1 levels were notably elevated, and LIAS and LIPT1 levels were significantly reduced in septic shock samples compared to normal controls (Fig 2A). The same result was found in the GSE33118 cohort (Fig 2B). Next, ROC curve was used to evaluate the diagnosis value of SLC31A1, MTF1, LIAS and LIPT1 in septic shock in the GSE26440 and GSE33118 cohorts. The AUC of ROC curves for these genes were 0.788, 0.908, 0.749 and 0.914 in the GSE26440 cohort, respectively (Fig 2C). Meanwhile, the AUC of ROC curves for SLC31A1, MTF1, LIAS and LIPT1 genes were 0.854, 0.983, 0.774 and 0.936 in the GSE33118 cohort, respectively (Fig 2D). These data suggested that SLC31A1, MTF, LIAS and LIPT1 may have potential diagnostic value in septic shock. Furthermore, GSE236713 cohort was utilized to verify SLC31A1, MTF1, LIAS and LIPT1 level in sepsis. Similarly, SLC31A1 and MTF1 levels were obviously elevated, and LIAS and LIPT1 levels were reduced in pulmonary sepsis samples compared to normal controls (S2A–S2D Fig).

Additionally, RT-qPCR was conducted to verify SLC31A1, LIAS, MTF1 and LIPT1 levels in septic shock patients. As shown in Fig 2E, compared to healthy controls, SLC31A1 and MTF1 levels were remarkably elevated, and LIAS and LIPT1 levels were notably decreased in peripheral blood lymphocytes from the septic shock patients.

Based on the median expression levels of SLC31A1, LIAS, MTF1 or LIPT1, septic shock samples in the GSE26440 cohort were divided into high- and low-level groups for each gene: high-SLC31A1, high-LIAS, high-MTF1 or high-LIPT1 level group and low-SLC31A1, low-LIAS, low-MTF1 or low-LIPT1 level group. A total of 2047 DEGs were identified between high- and low-SLC31A1 groups; 2658 DEGs were screened between high- and low-MTF1 groups; 2400 DEGs were screened between high- and low-LIAS groups; 1861 DEGs were screened between high- and low-LIPT1 groups (S3 Table).

Top 50 upregulated and top 50 downregulated genes were screened from both high-level and low-level groups. These genes were then input into the CMap database to predict potential drugs targeting them (S4 Table). Top 10 potential drugs for the treatment of septic shock were listed in Table 1. Given that SLC31A1 and MTF1 levels were elevated, and LIAS and LIPT1 levels were reduced in septic shock samples (Fig 2A and 2B), patients in the high-SLC31A1 and high-MTF1 groups may benefit from negatively-correlated drugs or molecules (negative score), whereas patients in the low-LIAS and low-LIPT1 groups may benefit from the positively correlated drugs or molecules (positive score) (Table 1).

**Table 1:** Top 10 potential drugs for septic shock treatment.

Gene	Rank	Score	ID	Name	Description
SLC31A1	28	95.07	BRD-K28428262	brivanib	FGFR inhibitor
	27	95.53	BRD-K34415467	trimethobenzamide	Histamine receptor antagonist
	22	95.92	BRD-K48722833	iloperidone	Dopamine receptor antagonist
	20	96.82	BRD-K02607075	tubocurarine	Acetylcholine receptor antagonist
	17	97.07	BRD-K29582115	ziprasidone	Dopamine receptor antagonist
	13	97.57	BRD-K64857848	XMD-885	Leucine rich repeat kinase inhibitor
	32	94.78	BRD-K50388907	fenofibrate	PPAR receptor agonist
	8528	-93.33	BRD-A14798026	mestranol	Estrogen receptor agonist
	8534	-94.62	BRD-K09638361	SA-63133	-666
	8537	-95.21	BRD-K92991072	PAC-1	Caspase activator
Gene	Rank	Score	ID	Name	Description
MTF1	35	96.76	BRD-K04111260	raclopride	Dopamine receptor antagonist
	51	94.92	BRD-A78877355	nefopam	Cyclooxygenase inhibitor
	59	94.57	BRD-A25576662	streptozotocin	DNA alkylating agent
	61	94.41	BRD-A85860691	chaetocin	Histone lysine methyltransferase inhibitor
	74	93.31	BRD-K56700933	phenethyl-isothiocyanate	Antineoplastic
	81	92.98	BRD-K81916719	triclabendazole	Microtubule inhibitor
	83	92.54	BRD-K81783531	VX-222	HCV inhibitor
	8543	-92.25	BRD-K31542390	mycophenolic-acid	Dehydrogenase inhibitor
	8545	-92.78	BRD-K95402279	geranylgeraniol	Farnesyltransferase inhibitor
	8548	-93.88	BRD-K40742111	baeomycesic-acid	Lipoxygenase inhibitor
Gene	Rank	Score	ID	Name	Description
LIAS	9	99.05	BRD-K21971034	OM-137	Aurora kinase inhibitor
	22	97.36	BRD-K33483813	actarit	Interleukin receptor agonist
	8531	-97.53	BRD-A39646320	HC-toxin	HDAC inhibitor
	8532	-97.57	BRD-K13810148	givinostat	HDAC inhibitor
	8540	-97.87	BRD-K81418486	vorinostat	HDAC inhibitor
	8542	-97.93	BRD-A80960055	celastrol	Anti-inflammatory
	8543	-97.99	BRD-K56957086	dacinostat	HDAC inhibitor
	8547	-98.48	BRD-K74761218	WT-171	HDAC inhibitor
	8549	-98.59	BRD-K69840642	ISOX	HDAC inhibitor
	8550	-98.77	BRD-K12867552	THM-I-94	HDAC inhibitor
Gene	Rank	Score	ID	Name	Description
LIPT1	8	98.64	BRD-K28143534	cyproheptadine	Histamine receptor antagonist
	10	97.92	BRD-K90864987	cobalt(II)-chloride	HSP inducer
	12	96.76	BRD-K57926513	tyrphostin-AG-1295	PDGFR receptor inhibitor
	16	95.65	BRD-K02607075	tubocurarine	Acetylcholine receptor antagonist
	21	94.62	BRD-K91696562	orantinib	FGFR inhibitor
	24	94.36	BRD-A62184259	cycloheximide	Protein synthesis inhibitor
	8529	-94.92	BRD-K04466929	Merck60	HDAC inhibitor
	8544	-97.71	BRD-K63265447	docetaxel	Tubulin inhibitor
	8553	-99.01	BRD-K11558771	droxinostat	HDAC inhibitor
	8556	-99.22	BRD-A65013509	oxybutynin	Acetylcholine receptor antagonist

### 3.3 Biological function analysis of hub genes in septic shock

**Fig 3:**
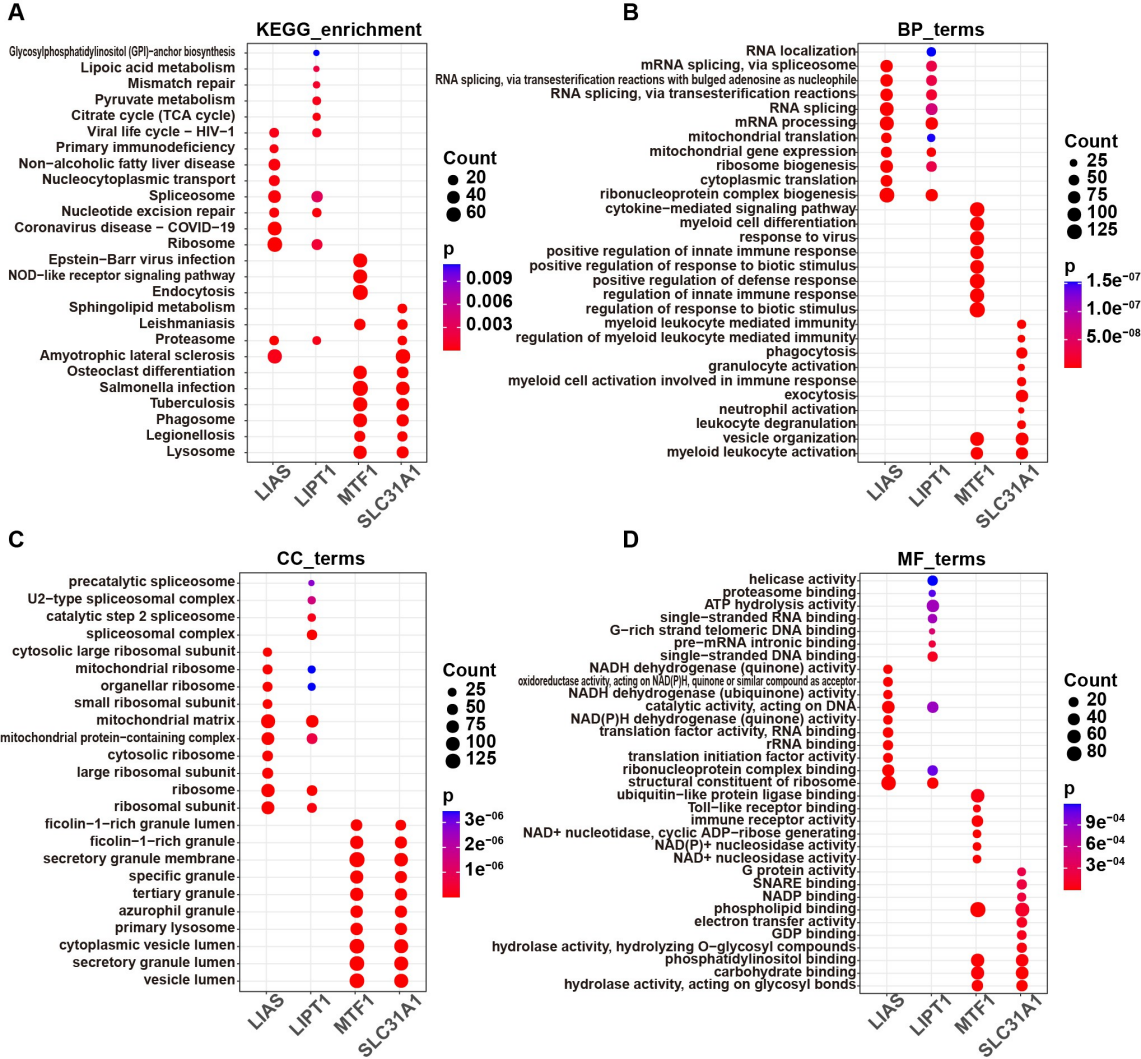
GO and KEGG enrichment analyses of hub genes in septic shock. **(A)** Top 10 KEGG enrichment pathways, **(B)** top 10 GO-BP terms, **(C)** top 10 GO-CC terms and **(D)** top 10 GO-MF terms of the DEGs between high-SLC31A1, -MTF1, -LIAS or -LIPT1 level group and low-SLC31A1, -MTF1, -LIAS or -LIPT1 level group respectively.

According to the DEGs listed in the S3 Table, GO and KEGG analyses were performed. The DEGs between high- and low-SLC31A1 groups were enriched in 88 KEGG pathways, 1138 GO-BP terms, 57 GO-MF terms and 23 GO-CC terms (S5 and S6 Tables). The DEGs between high- and low-MTF1 level groups were enriched in 95 KEGG pathways, 1546 GO-BP terms, 195 GO-MF terms and 177 GO-CC terms (S5 and S6 Tables). The DEGs between high- and low-LIAS level groups were enriched in 48 KEGG pathways, 677 GO-BP terms, 127 GO-MF terms and 148 GO-CC terms (S5 and S6 Tables). The DEGs between high- and low-LIPT1 level groups were enriched in 27 KEGG pathways, 616 GO-BP terms, 90 GO-MF terms and 115 GO-CC terms (S5 and S6 Tables). The top 10 enriched terms in GO-BP, GO-MF and GO-CC categories, as well as the top 10 enriched KEGG pathways were displayed in Fig 3A–3D.

**Fig 4:**
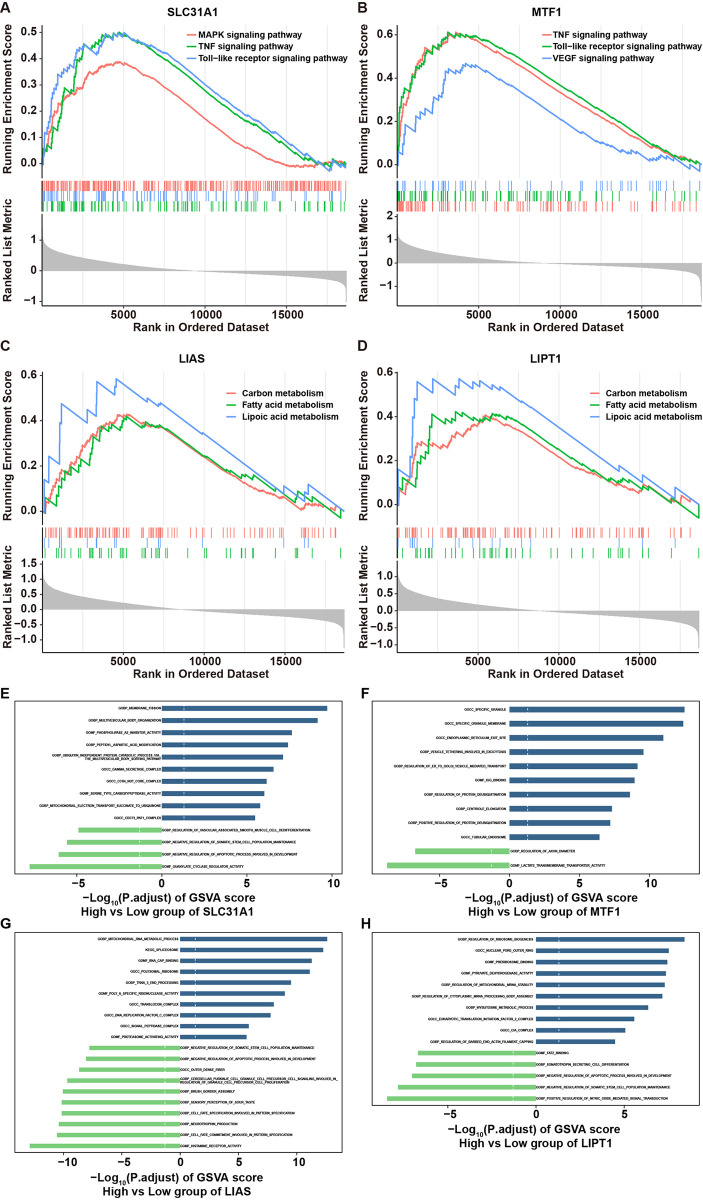
GSEA and GSVA analyses of hub genes in septic shock. **(A, B, C, D)** GSEA results between high-SLC31A1, -MTF1, -LIAS or -LIPT1 level group and low-SLC31A1, -MTF1, -LIAS or -LIPT1 level group respectively. **(E, F, G, H)** GSVA results between high-SLC31A1, -MTF1, -LIAS or -LIPT1 level group and low-SLC31A1, -MTF1, -LIAS or -LIPT1 level group respectively.

Furthermore, gene functional enrichment was then performed by GSEA and GSVA. The GSEA results showed that compared to low-SLC31A1 group, 163 pathways, including TNF signaling, toll−like receptor signaling, MAPK signaling pathways, were notably enriched in the high-SLC31A1 group (Fig 4A and S7 Table). Compared to low-MTF1 group, 170 pathways, including TNF signaling, toll−like receptor signaling, and VEGF signaling pathways, were enriched in the high-MTF1 group (Fig 4B and S7 Table). Compared to low-LIAS or low-LIPT1 group, 149 and 142 pathways, respectively, including fatty acid metabolism, lipoic acid metabolism and carbon metabolism pathways, were enriched in high-LIAS or high-LIPT1 group (Fig 4C and 4D and S7 Table).

Meanwhile, GSVA results showed that DEGs between high- and low-SLC31A1 groups were involved in 28 pathways (e. g. regulation_of_helicase_activity and mitochondrial_DNA_replication); DEGs between high- and low-MTF1 groups were involved in 107 pathways (e. g. neutrophil_degranulation and regulation_of_neutrophil_activation); DEGs between high- and low-LIAS groups were involved in 230 pathways (e. g. tDNA_methyltransferase_activity and meiotic_telomere_clustering); DEGs between high- and low-LIPT1 groups were involved in 47 pathways (e. g. uniplex_complex and 7_methylguanosine_RNA_capping) (Fig 4E–4H and S8 Table).

### 3.4 Evaluation of immune infiltration between control and septic shock samples

**Fig 5:**
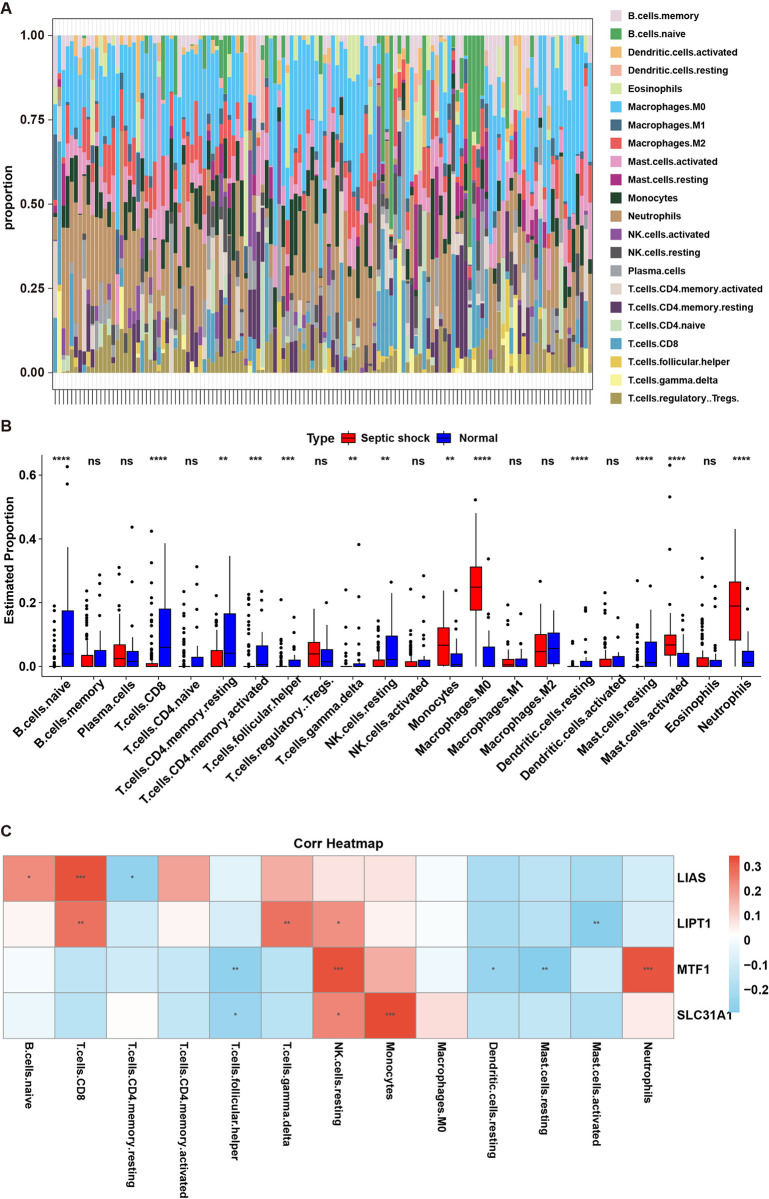
Evaluation of immune infiltration between control and septic shock samples. **(A)** Bar plot displayed the proportions of the 22 types of infiltrated immune cells in each sample. **(B)** The Box plots displayed the proportions of 22 immune cells between control and septic shock groups. **(C)** The correlation between SLC31A1, MTF1, LIAS or LIPT1 level and immune cells.

Based on the data from GSE26440 cohort, the relative level of 22 immune cells in each sample was evaluated using the CIBERSORT algorithm (Fig 5A). Meanwhile, the proportions of 13 immune cells including naive B cells, CD8 T cells, resting CD4 memory T cells, activated CD4 memory T cells, follicular helper T cells, gamma delta T cells, resting NK cells, monocytes, M0 macrophages, resting dendritic cells, resting mast cells, activated mast cells and neutrophils were significantly altered between control and septic shock groups (Fig 5B). Moreover, SLC31A1 gene was positively correlated with the proportions of resting NK cells and monocytes and was negatively correlated with follicular helper T cells levels; MTF1 gene was positively correlated with resting NK cells and neutrophils levels and was negatively correlated with follicular helper T cells, resting dendritic cells and resting mast cells levels; LIAS was positively correlated with naive B cells and CD8 T cells levels and was negatively correlated with resting CD4 memory T cells levels; LIPT1 was positively correlated with gamma delta T cells, resting NK cells and CD8 T cells levels and was negatively correlated with activated mast cells levels (Fig 5C).

### 3.5 Construction of hub gene-miRNAs-lncRNAs network and hub gene-TFs network

**Fig 6:**
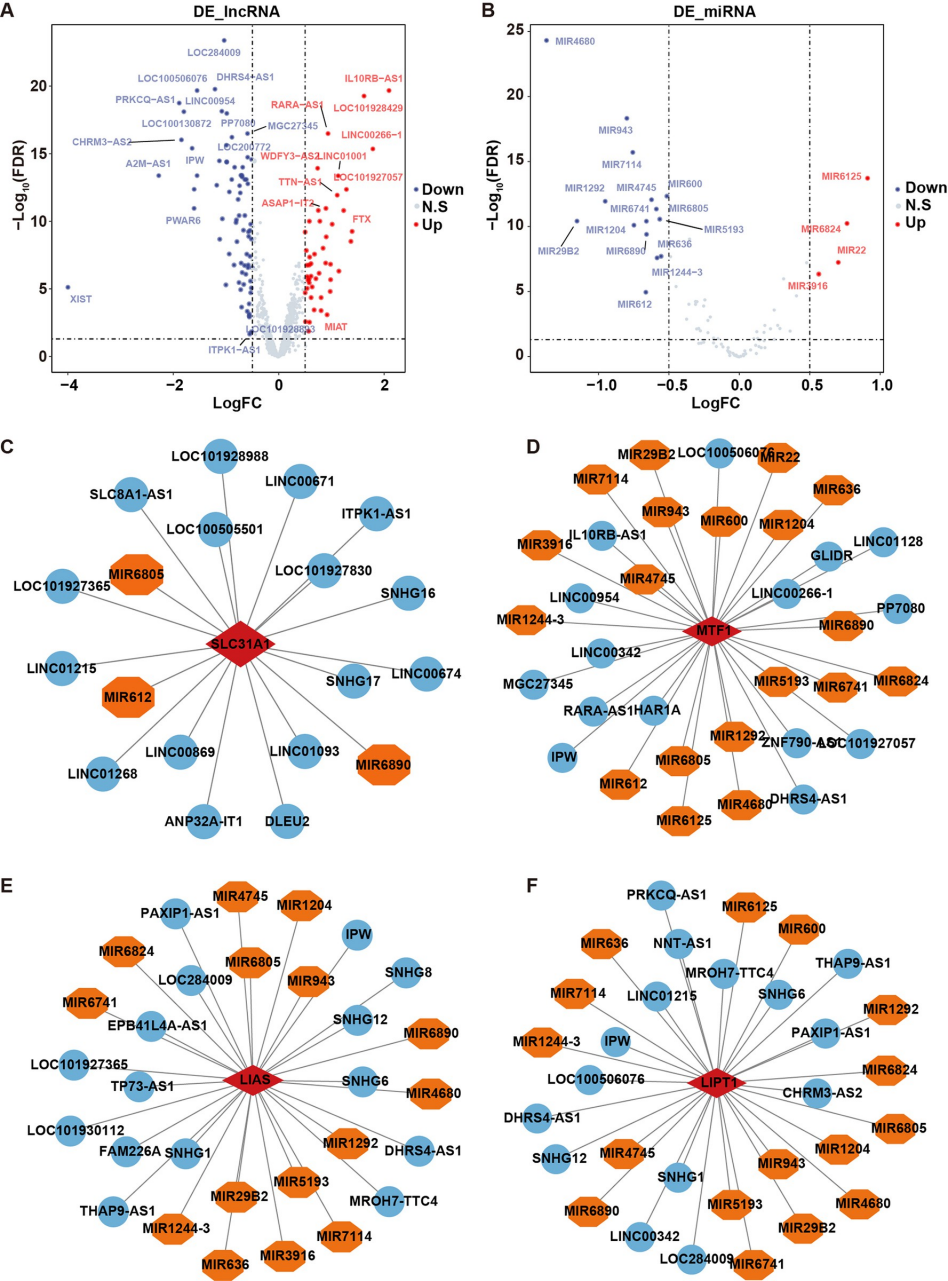
Construction of hub gene-miRNAs-lncRNAs network. **(A)** The volcano plot displayed the differential expressed lncRNAs between control and septic shock groups in the GSE57065 cohort. **(B)** The volcano plot displayed the differential expressed miRNAs between control and septic shock groups in the GSE57065 cohort. **(C)** The network constructed by SLC31A1 gene with 3 miRNAs and top 15 lncRNAs. **(D, E, F)** The networks constructed by MTF1, LIAS or LIPT1 gene with top 15 miRNAs and top 15 lncRNAs.

Based on the data from the GSE57065 cohort, differential expressed miRNAs (DEmiRNAs) and differential expressed lncRNAs (DElncRNAs) were identified between control and septic shock groups. The results showed that 123 DElncRNAs and 19 DEmiRNAs were screened between control and septic shock groups (Fig 6A and 6B and S9 Table). The correlation coefficients between hub genes and miRNAs or lncRNAs were calculated (S10 Table). Top 15 DElncRNAs and top 15 DEmiRNAs exhibiting the highest correlation with the SLC31A1, LIAS, MTF1 or LIPT1 gene were selected for network generation (Fig 6C–6F).

**Fig 7:**
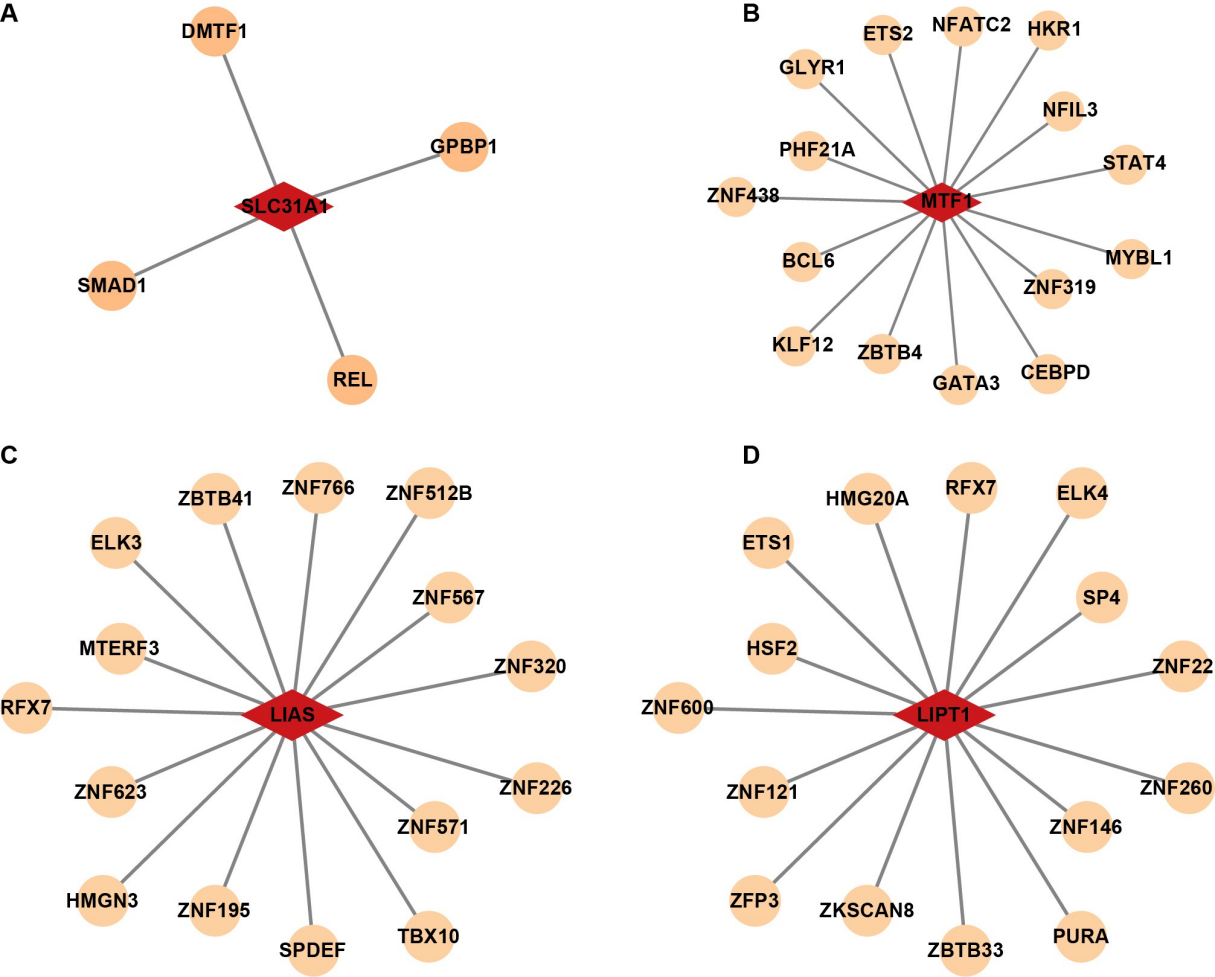
Construction of hub gene-TFs network. **(A)** The network constructed by SLC31A1 gene with 4 TFs. **(B, C, D)** The networks constructed by MTF1, LIAS or LIPT1 gene with top 15 TFs.

Furthermore, the correlation coefficients between hub genes and TFs were then calculated (S10 Table). Top 15 TFs with the highest correlation to the SLC31A1, LIAS, MTF1 or LIPT1 gene were also chosen for network generation (Fig 7A–7D).

## 4. Discussion

The early diagnosis of septic shock is essential for enhancing patient outcomes. Numerous studies have shown that specific blood parameters, such as C-reactive protein, procalcitonin and neutrophil to lymphocyte ratio, provide significant diagnostic and prognostic value for infectious diseases [32–35]. These biomarkers can assist clinicians in making timely decisions that ultimately improve the prognosis for patients with septic shock. Furthermore, recent research has identified some novel factors, such as Irisin and TNF-related apoptosis-inducing ligand (TRAIL), which also show promise for improving early diagnostic capabilities in septic shock [36,37]. The exploration of new biomarkers may lead to more effective diagnostic and therapeutic strategies in clinical settings. Cuproptosis, a novel form of cell death, has become the focus of research in recent years [38]. Evidence has shown that cuproptosis-related genes (e.g. PDXK, FDX1 and LIPT1) can affect the progression of human cancers [39–41]. Chang et al. identified FDX1 and LIPT1 as potential therapeutic targets for osteoarthritis [38]. Additionally, Liu et al. found that GLS had good diagnostic value in acute myocardial infarction [42]. However, the impacts of cuproptosis-related genes in septic shock have not been clearly demonstrated. In this study, we found that SLC31A1, LIAS, MTF1 and LIPT1 levels were dysregulated between control and septic shock samples. Meanwhile, two cohorts (GSE26440 and GSE33118) were utilized to evaluate the diagnosis value of SLC31A1, MTF1, LIAS and LIPT1 in septic shock. The AUC of ROC curves for MTF1 and LIPT1 genes were greater than 0.9 in two cohorts, suggesting that these two genes had good diagnostic performances [43]. Meanwhile, the AUC of ROC curves for SLC31A1 and LIAS genes were higher than 0.7 in two cohorts, suggesting that these two genes had acceptable diagnostic performances [43]. These results showed that these four genes had certain to good diagnostic performances.

SLC31A1, also known as copper transporter 1 (CTR1), is a key copper transporter [44]. Huo et al. found that advanced glycosylation end products could trigger cuproptosis in cardiomyocytes through upregulating SLC31A1 [45], suggesting that SLC31A1 may exert a pro-cuproptosis role. Additionally, MTF1 has been identified as playing a role in metal detoxification and maintaining metal redox homeostasis [46]. MTF1 can protect cells under high copper conditions through activating metallothioneins, while it also can obtain scarce copper from the surroundings upon copper starvation through upregulating Ctr1B (a copper importer) [47]. Evidence has shown that copper exposure can lead to toxicity and inflammatory response in zebrafish larvae [48]. Zhong et al. found that high concentrations of copper could induce liver lipotoxicity by enhancing Nrf2 transcriptional activation via MTF1 activation [49]. Hao et al. found that miR-22 could induce inflammatory pain in dorsal horn neurons via increasing Mtf1 and upregulating p-ERK1/2, GFAP, and c-Fos [50]. These findings showed that elevated levels of MTF1 may related to copper-induced cell damage. Furthermore, it has been shown that SLC31A1 and MTF1 levels, as well as serum copper levels, were all elevated in psoriatic patients compared to controls [51,52], suggesting that SLC31A1 and MTF1 may be risk factors for psoriasis. Consistent with previous studies, SLC31A1 and MTF1 were significantly elevated in septic shock samples compared to normal controls. These data showed that SLC31A1 and MTF1 may serve as biomarkers for septic shock.

Furthermore, Balamurugan et al. found that MTF-1 could promote the transcription of PIGF, an angiogenic gene [53]. Vascular barrier breakdown has been observed in patients with sepsis, as evidenced by the upregulation of angiogenic-related gene (e.g. VEGF-A) [54]. Moreover, the interaction between PIGF and VEGF plays a crucial role in modulating angiogenesis and vascular permeability [55]. In the current research, the GSEA results showed that compared to low-MTF1 groups, VEGF signaling pathway was more activated in high-MTF1 group. Thus, MTF1 may participate in the progression of septic shock by affecting angiogenesis and vascular permeability. Additionally, SLC31A1 (CTR1) has been shown to activate MAPK signaling [56,57], which has been implicated in the progression of septic shock [58]. Inhibiting the activation of MAPK signaling could attenuate organ injury in mice with septic shock [59,60]. In this study, GSEA results showed that compared to low-MTF1 groups, MAPK signaling pathway was more activated in high-MTF1 group. Thus, SLC31A1 may promote the progression of septic shock through activating MAPK signaling. However, these assumptions require further investigation in future research.

LIAS is an iron-sulfur cluster protein that can catalyze the final step of the biosynthesis of lipoic acid [61]. Meanwhile, LIPT1 is also a key enzyme involving in lipoic acid biosynthesis [62,63]. Lipoic acid acts as an antioxidant, which has been found to improve immune dysfunction and organ injury in sepsis [64]. Nie et al. found that inhibiting YY1/lipoic acid pathway could enhance cuproptosis in ovarian cancer stem cells [65]. In the current research, the GSEA results showed that compared to low-level groups, lipoic acid metabolism pathway was more activated in high-LIAS or high-LIPT1 group. These findings demonstrated a potential relationship among LIAS and LIPT1, lipoic acid pathway and cuproptosis in septic shock. Cui et al. reported elevated copper ions and reduced LIPT1 levels in plaques of atherosclerotic patients [66]. Moreover, compared to healthy controls, serum copper levels were higher, but LIPT1 levels were lower in patients with Crohn’s disease [67,68]. Meanwhile, Liu et al. found that compared to stable coronary heart disease, LIAS and LIPT1 were notably reduced in AMI and suggested that LIAS and LIPT1 may serve as protective factors for AMI [42]. Consistent with previous studies, LIAS and LIPT1 were notably reduced in septic shock samples compared to normal controls. These data showed that LIAS and LIPT1 may serve as protective factors for septic shock. However, these hypotheses require further investigation in future studies.

It has been shown that immune dysregulation is prevalent among patients with septic shock [69,70]. For example, the function and number of CD8+ T cells were diminished in patients with sepsis [71]. Moreover, compared to the sham group, the numbers of CD4+ and CD8+ T cells were reduced and Treg cells were increased in the spleens of a septic rat model, suggesting an immunosuppressive status in septic rats [72]. Xia et al. discovered that elevating the levels of splenic CD4+ and CD8+ T cells could improve the survival rate of mice with septic immunosuppression [73]. In the present study, we found that LIAS and LIPT1 exhibited a positive correlation with CD8+ T cells, suggesting that LIAS and LIPT1 may contribute to improving immune status in septic shock patients by elevating the number of CD8+ T cells. Additionally, NK cells have been implicated in the pathogenesis of septic shock [74]. Excessive NK cell activation could enhance inflammatory response during sepsis, eventually leading to organ injury [75]. Our data showed that SLC31A1 and MTF1 exhibited a positive correlation with NK cells, suggesting that SLC31A1 and MTF1 may facilitate the progression of septic shock though activating NK cells; however, this hypothesis requires further investigation in future studies. Evidence has shown that T cells play a crucial role in the adaptive immune response, while NK cells are instrumental in regulating the innate immune response [76,77]. Thus, we suggest that these four genes may be associated with the host immune response in septic shock. Additionally, research indicates that the occurrence of infectious diseases, including septic shock, COVID-19 and influenza, is closely linked to the host’s immune response, which encompasses both innate and adaptive immune systems [78–81]. Hence, we propose an idea that these genes may have diagnostic potential for other infectious diseases, such as COVID-19. However, this hypothesis needs to be further investigated in future studies.

## 5. Conclusion

In this study, we found that the levels of SLC31A1 and MTF1 were obviously upregulated and the levels of LIAS and LIPT1 were downregulated in septic shock samples. These genes exhibited potential diagnostic efficacy for septic shock. These results showed that SLC31A1, MTF1, LIAS and LIPT1 may serve as potential biomarkers for the diagnosis of septic shock. However, the function of SLC31A1, MTF1, LIAS and LIPT1 in septic shock remain unexplored. As a significant contributor to sepsis and septic shock, lipopolysaccharide (LPS) has been examined in both *in vivo* and *in vitro* settings [82]. Consequently, investigating the roles of these genes in septic shock could enhance our understanding of its pathogenesis. Thus, future research should integrate *in vitro* assays with *in vivo* animal models to investigate the roles of four genes in septic shock and to elucidate the underlying mechanisms.

## Supporting information

S1 Fig The expression of four genes in human tissues. **(A-D)** The expression distribution of SLC31A1, MTF1, LIAS and LIPT1 genes in human tissues was analyzed by The Human Protein Atlas (HPA) database.
(TIF)

S2 Fig The expression levels of four genes between sepsis and control samples in the GSE236713 cohort. **(A-D)** The Box plots displayed SLC31A1, MTF1, LIAS and LIPT1 levels between control and sepsis groups in the GSE236713 cohort.
(TIF)

S1 Table 19 cuproptosis-related genes. (XLSX)

S2 Table The clinical information of participants. (XLSX)

S3 Table DEGs between high hub gene level group and low hub gene level group. (XLSX)

S4 Table Potential drugs for septic shock treatment. (XLSX)

S5 Table KEGG enrichment pathways. (XLSX)

S6 Table GO terms. (XLSX)

S7 Table GSEA enrichment pathways. (XLSX)

S8 Table GSVA enrichment pathways. (XLSX)

S9 Table Differential expressed lncRNAs and miRNAs between control and septic shock groups. (XLSX)

S10 Table The correlation of each hub gene and lncRNAs, miRNAs or TFs. (XLSX)
