# Supplementary figures and images for: Identification of cuproptosis-related genes in septic shock based on bioinformatic analysis
Source: PLoS One. 2024 Dec 9;19(12):e0315219. doi: 10.1371/journal.pone.0315219 (PMC11627398; doi:10.1371/journal.pone.0315219)

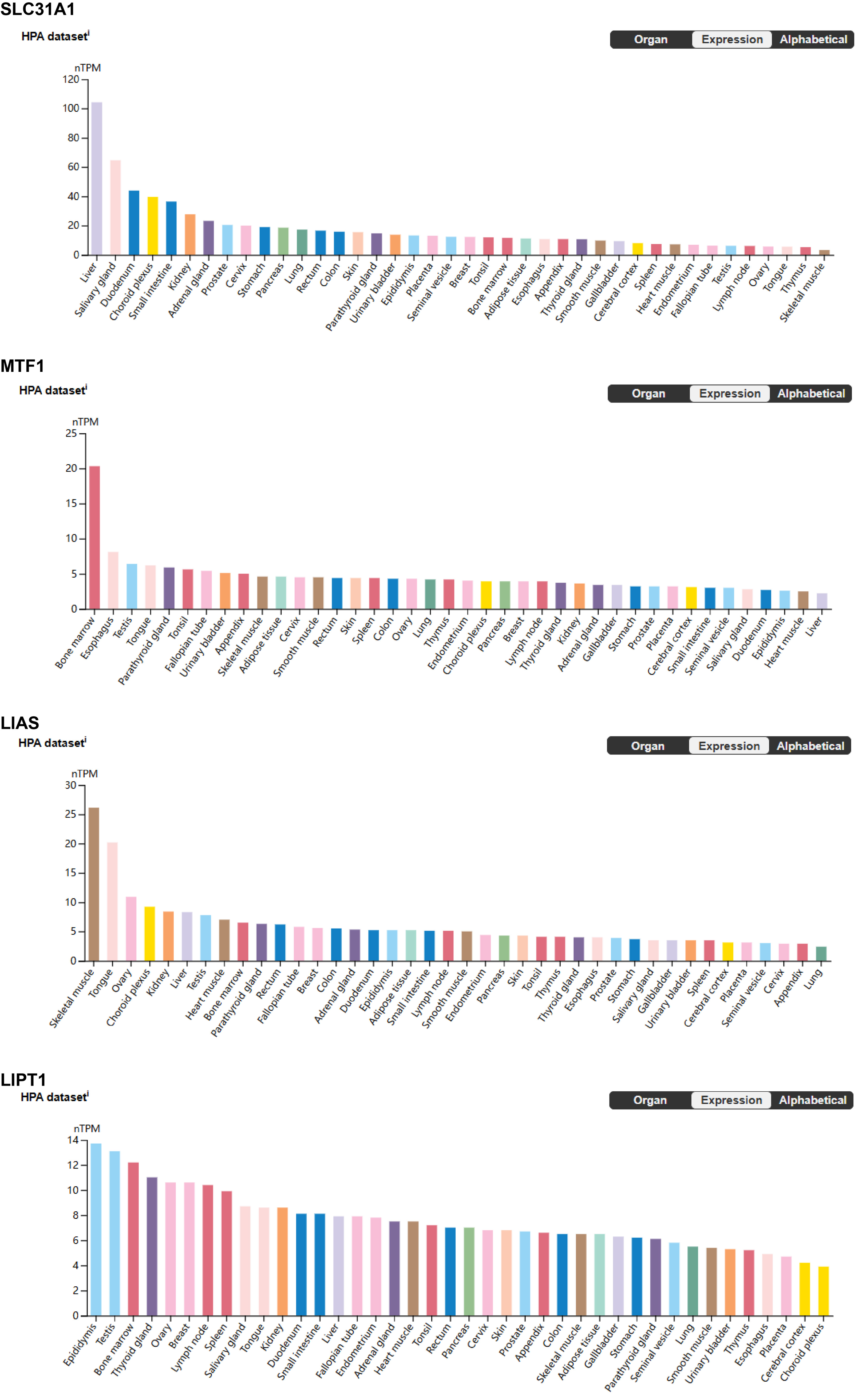

Supplement: S1 Fig — (A-D) The expression distribution of SLC31A1, MTF1, LIAS and LIPT1 genes in human tissues was analyzed by The Human Protein Atlas (HPA) database. (TIF) [file pone.0315219.s001.tif]

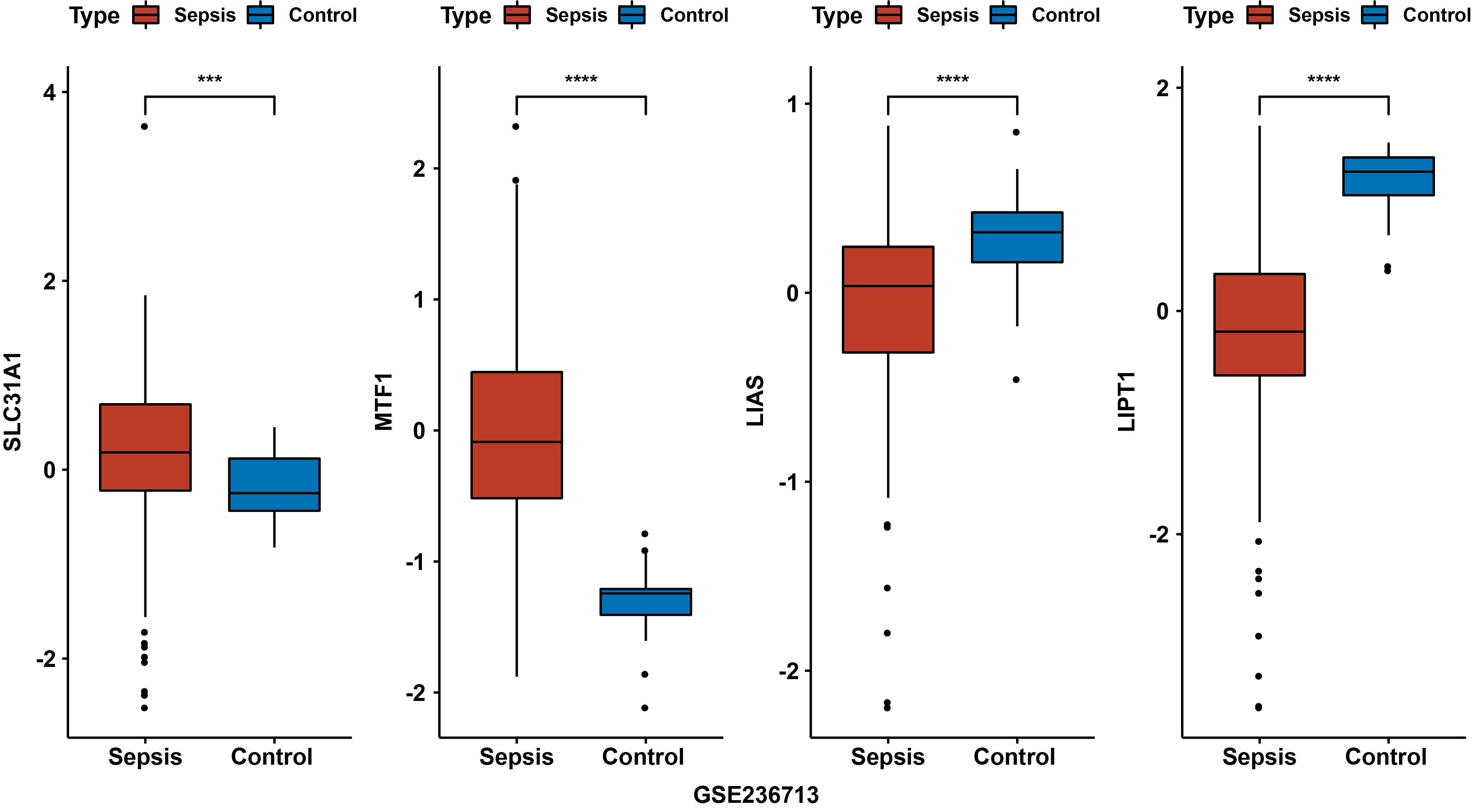

Supplement: S2 Fig — (A-D) The Box plots displayed SLC31A1, MTF1, LIAS and LIPT1 levels between control and sepsis groups in the GSE236713 cohort. (TIF) [file pone.0315219.s002.tif]
